# MODIS-Based Spatiotemporal Inversion and Driving-Factor Analysis of Cloud-Free Vegetation Cover in Xinjiang from 2000 to 2024

**DOI:** 10.3390/s25082394

**Published:** 2025-04-09

**Authors:** He Yang, Min Xiong, Yongxiang Yao

**Affiliations:** 1 School of Remote Sensing and Information Engineering, Wuhan University, Wuhan 430079, China; yheyhe@whu.edu.cn; 2 Army Infantry Academy, Nanchang 330000, China; 18279215083@163.com

**Keywords:** cloud removal, MODIS remote sensing image, fractional vegetation cover, spatiotemporal variation, driving factors

## Abstract

The Xinjiang Uygur Autonomous Region, characterized by its complex and fragile ecosystems, has faced ongoing ecological degradation in recent years, challenging national ecological security and sustainable development. To promote the sustainable development of regional ecological and landscape conservation, this study investigates Fractional Vegetation Cover (FVC) dynamics in Xinjiang. Existing studies often lack recent data and exhibit limitations in the selection of driving factors. To mitigate the issues, this study utilized Google Earth Engine (GEE) and cloud-free MOD13A2.061 data to systematically generate comprehensive FVC products for Xinjiang from 2000 to 2024. Additionally, a comprehensive and quantitative analysis of up to 15 potential driving factors was conducted, providing an updated and more robust understanding of vegetation dynamics in the region. This study integrated advanced methodologies, including spatiotemporal statistical analysis, optimized spatial scaling, trend analysis, and Geographical Detector (GeoDetector). Notably, we propose a novel approach combining a Theil–Sen Median trend analysis with a Hurst index to predict future vegetation trends, which to some extent enhances the persuasiveness of the Hurst index alone. The following are the key experimental results: (1) Over the 25-year study period, Xinjiang’s vegetation cover exhibited a pronounced north–south gradient, with significantly higher FVC in the northern regions compared to the southern regions. (2) A time series analysis revealed an overall fluctuating upward trend in the FVC, accompanied by increasing volatility and decreasing stability over time. (3) Identification of 15 km as the optimal spatial scale for FVC analysis through spatial statistical analysis using Moran’s I and the coefficient of variation. (4) Land use type, vegetation type, and soil type emerged as critical factors, with each contributing over 20% to the explanatory power of FVC variations. (5) To elucidate spatial heterogeneity mechanisms, this study conducted ecological subzone-based analyses of vegetation dynamics and drivers.

## 1. Introduction

In recent years, the Chinese government has attached great importance to the construction of ecological civilization [1,2] and implemented a series of ecological restoration policies. The Xinjiang Uygur Autonomous Region, situated in the arid northwest of China, is characterized by complex geomorphological features and significant bioclimatic differentiation between its northern and southern regions. With its vulnerable ecological environment [3], this region serves as a crucial ecological security barrier, while simultaneously presenting challenges to sustainable economic development.

Fractional Vegetation Cover (FVC) is defined as the proportion of the vertical projection area of vegetation within a unit area relative to the total area [4], which is a key parameter for ecosystem evaluation [5]. Remote sensing methods for FVC estimation include regression models, machine learning techniques, mixed-pixel decomposition, and dimidiate pixel model. (1) Regression models [6,7] employ mathematical statistics to establish relationships between remotely sensed spectral bands or vegetation indices and measured FVC values. While effective, these methods rely on extensive field-measured data and are subject to subjective model selection, limiting their generalizability. (2) Machine learning methods [8,9] train models using measured FVC and auxiliary data, improving both accuracy and generalization. (3) Mixed-pixel decomposition [10,11] decomposes pixels into a linear combination of information from different land cover types to estimate endmember abundances. It is particularly suitable for regions with complex land cover. However, its effectiveness is significantly limited by the selection and quantity of endmembers. (4) Dimidiate pixel model [12,13], grounded in mixed-pixel decomposition, uses data from fully vegetated and bare soil pixels to construct an inversion model. This method effectively reduces the influence of soil on FVC inversion and does not require field-measured FVC data, offering high adaptability and generalization.

Google Earth Engine (GEE), an open-source cloud-computing platform for geospatial analysis [14], provides advanced processing capabilities and comprehensive remote sensing datasets [15]. Given the requirements for large-scale, long-term, multi-scale, and high-precision FVC estimation in Xinjiang, GEE’s cloud-computing infrastructure and integrated data resources enable efficient processing of massive geospatial data. Therefore, this study utilizes the dimidiate pixel model implemented on the GEE platform for FVC inversion. This approach can optimally balance computational efficiency with inversion accuracy while effectively addressing mixed-pixel challenges in arid environments.

Numerous studies have investigated the driving mechanisms of FVC dynamics through quantitative analysis of multiple influencing factors. Climatic variables, particularly temperature and precipitation, have been identified as dominant natural drivers of FVC variations [16]. The study by Ma et al. [17] emphasizes the need for caution when applying vegetation indices in mountainous areas and advocates for thorough consideration of topographic effects. Concurrently, anthropogenic activities, including land use change and urbanization, have significantly impacted vegetation dynamics at regional scales [18]. Recent studies have increasingly adopted integrated approaches that incorporate both natural and anthropogenic factors [19,20,21], enabling more comprehensive analysis of vegetation cover dynamics and their underlying mechanisms. To fully understand FVC evolution and its driving forces, integrated and quantitative analytical approaches are essential. Such methods combine diverse datasets, quantify factor contributions, and uncover interaction mechanisms [22], providing valuable support for regional ecological planning and decision-making.

However, recent studies on vegetation analysis in Xinjiang have been relatively scarce and are insufficient to meet the current urgent demands of green ecological development in the region. Additionally, data availability constraints have hindered comprehensive analyses of driving factors. This study quantitatively investigates the long-term spatiotemporal dynamics of vegetation cover in Xinjiang from 2000 to 2024. An integrated methodological framework was adopted, combining trend analysis, stability analysis, correlation analysis, and the GeoDetector1.0-5 tool to conduct a comprehensive, multi-dimensional assessment of vegetation dynamics in the region.

In summary, this study innovatively integrated trend analysis, geographical detector, and correlation analysis to quantitatively reveal the spatiotemporal evolution characteristics of vegetation cover and its driving mechanisms in Xinjiang from 2000 to 2024 and added ecological sub-regional analysis. The findings can not only offer an objective evaluation and feedback on the implementation effectiveness of major national ecological restoration projects but also provide optimization strategies for the precise implementation of subsequent ecological restoration initiatives, such as the sixth phase of the “Three-North Shelterbelt Program”. These contributions directly support national ecological civilization construction and the “Dual Carbon” strategic goals. Additionally, the technical framework established in this study can serve as a robust reference paradigm for arid ecosystem management and national ecological security barrier construction.

## 2. Materials and Methods

### 2.1. Study Area

**Figure 1:**
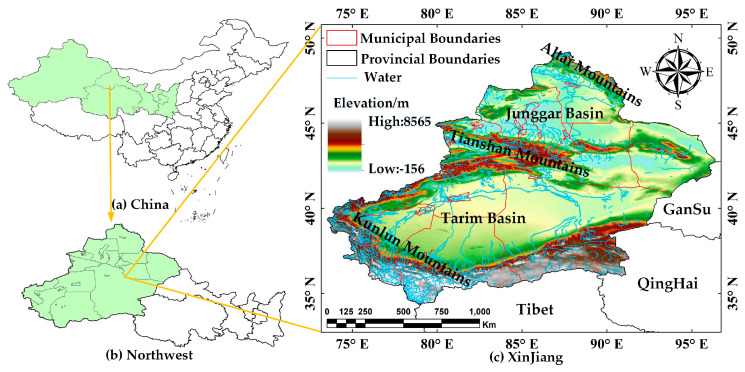
Location of the study area: (**a**) China, (**b**) Northwest, (**c**) XinJiang.

This study focuses on Xinjiang, located in northwestern China (34.3° N–49.1° N, 73.5° E–96.3° E). The study area encompasses approximately 1.66 million km², representing about one-sixth of China’s total land area. The region features a distinctive topography of alternating mountains and basins [23], known as the “three mountains and two basins” structure. From north to south, the region consists of the Altai Mountains, Junggar Basin, Tianshan Mountains, Tarim Basin, and Kunlun Mountains [24] (Figure 1) (Map of China from Tiantutu). Xinjiang is encircled by high mountains and situated far from the oceans, which prevents water vapor from entering the basins [25], resulting in a typical temperate continental climate. These geographical and climatic conditions contribute to a dry climate characterized by significant temperature fluctuations, low precipitation, and high evapotranspiration [26]. As one of China’s primary arid regions and a critical part of Central Asia’s arid zone [27], Xinjiang faces considerable challenges to vegetation growth [28].

In addition, central Xinjiang’s oasis zones serve as hubs for population and economic activities, making vegetation in this area heavily influenced by human activities. In recent years, climate change has increased the frequency of extreme weather events, which, combined with human factors such as overgrazing and land reclamation, have worsened ecological issues in the region [29]. Thus, analyzing the spatiotemporal variations and driving factors of vegetation cover in Xinjiang is imperative. Such investigations enhance understanding of evolutionary mechanisms shaped by the interaction of natural conditions and human activities [30].

**Figure 2:**
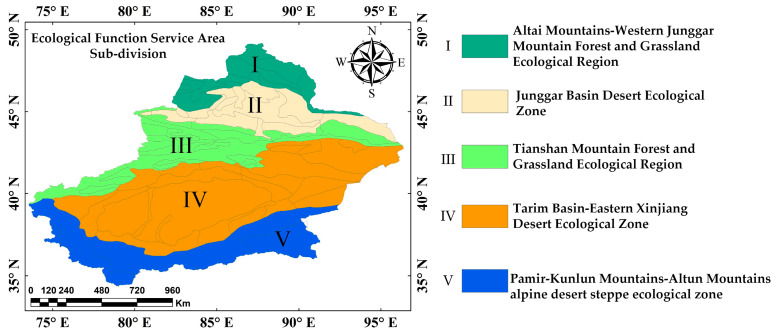
Ecological function service area sub-division of XinJiang.

To enhance the mechanistic understanding, this study incorporates a sub-regional ecological zoning framework, dividing the study area into five functionally distinct ecological sub-regions (Figure 2). Subsequent partitioned modeling was employed to quantitatively analyze the spatial heterogeneity and its driving factors.

### 2.2. Data Sources and Processing

Studies have shown a strong correlation between the Normalized Difference Vegetation Index (NDVI) and vegetation cover [31,32]. Therefore, this study used MOD13A2.061 remote sensing data with a 1 km spatial resolution and 16-day temporal resolution to obtain NDVI values. Xinjiang is a mountainous region with more anomalies in terrain data in winter [33] and more clouds/snow in cold seasons. So, to ensure optimal data quality, this study focused on the period from June to August for data computation and fusion. Meanwhile, cloud removal was performed using the DetailedQA bitmask, which filters out clouds, snow/ice, and shadows while retaining only high-quality pixels. This preprocessing step can enhance the reliability of vegetation measurements.

When selecting driving factors, we prioritized scientific relevance, representativeness, and quantifiability, considering both natural and human factors. Based on these considerations, a total of 15 driving factors were selected, 10 natural factors and 5 human factors. To ensure temporal consistency, all data with strong temporal variability, such as annual average temperature, annual precipitation, and nighttime light, were specifically chosen for the year 2020. Additionally, to address spatial scale discrepancies, all driving factor datasets were resampled to the optimal spatial scale determined for subsequent analysis. This approach can harmonize both temporal and spatial dimensions, minimizing discrepancies in data coverage. Consequently, the final dataset can provide consistent representativeness and support robust statistical analysis.

**Figure 3:**
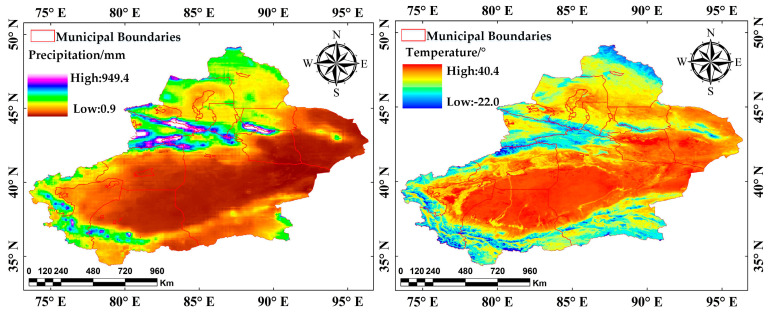

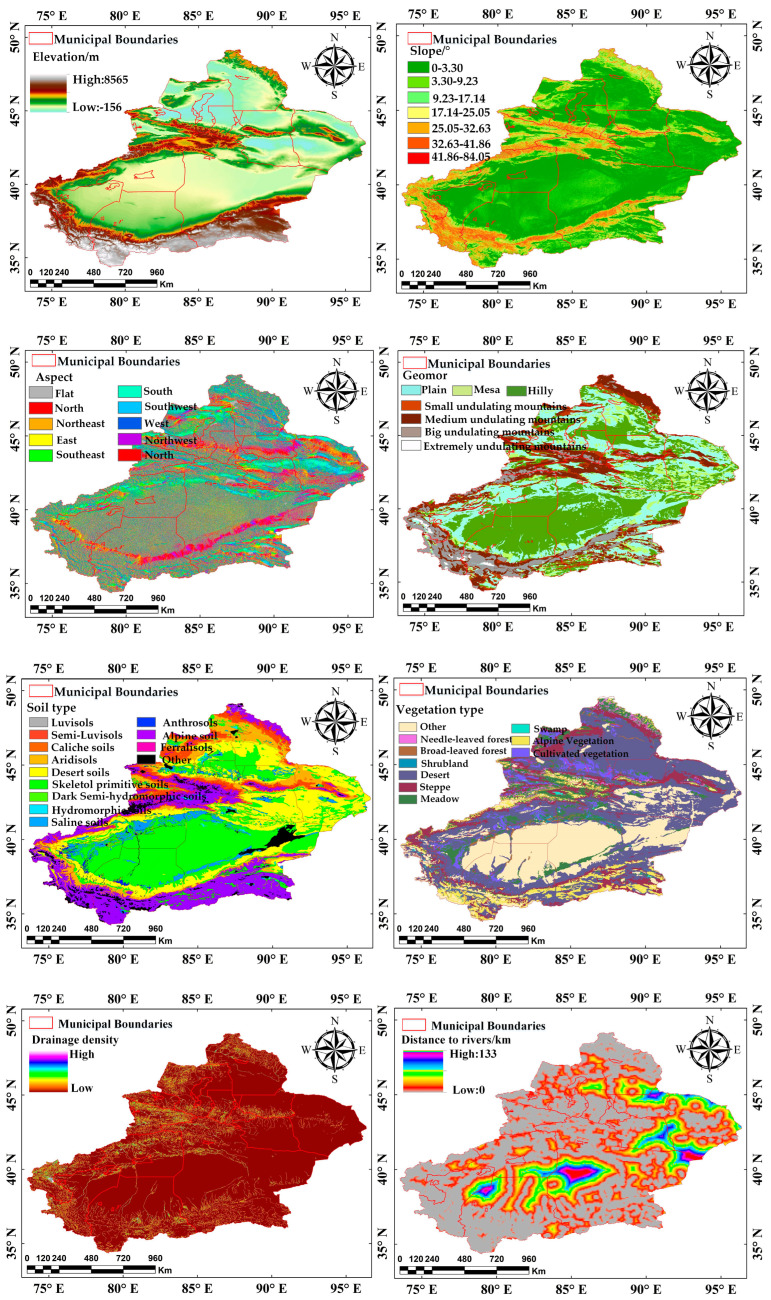

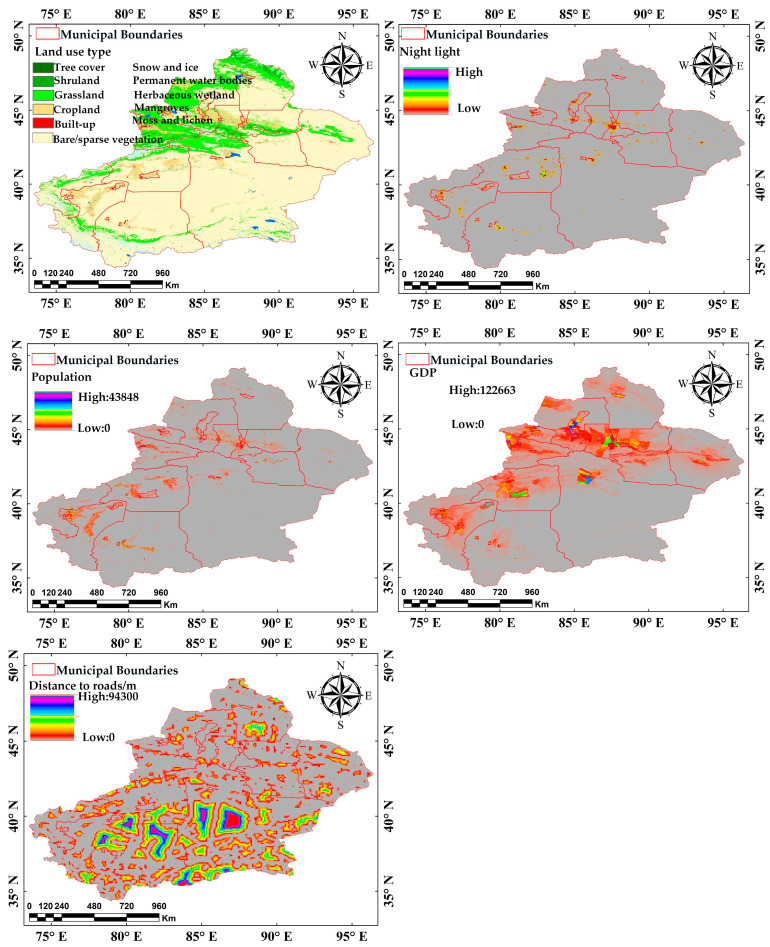
Spatial distribution of 15 driving factors.

Elevation data from the Copernicus DEM GLO30 dataset and slope and aspect were derived from the DEM data using the surface-fitting method and were included as driving factors. The slope affects soil moisture and runoff, while the aspect influences solar radiation and microclimate, both impacting vegetation growth. Including these variables allows for a more accurate interpretation of spatial vegetation patterns. Climate data were sourced from the GEE. Geomorphology, soil, and vegetation data were obtained from https://www.resdc.cn/ (accessed on 12 November 2024), while drainage density data came from https://cstr.cn/31253.11.sciencedb.j00001.00759 (accessed on 12 November 2024) [34]. Euclidean distances to rivers and roads were calculated in ArcGIS using road and hydrological data from OpenStreetMap (OSM). Additional datasets, such as geomorphology, nighttime light, and population density, are detailed in Table 1. The data of each driving factor is shown in Figure 3.

### 2.3. Methods

#### 2.3.1. Dimidiate Pixel Model

The FVC was calculated using the pixel dichotomy model, with *NDVI_veg_* and *NDVI_soil_* defined based on the study area’s NDVI histogram, using the 5th and 95th percentiles as thresholds.

(1) $$FVC_{i}=\frac{NDVI_{i}-{NDVI}_{soil}}{{NDVI}_{veg}-{NDVI}_{soil}}\times 100\%$$

The FVC was classified into five levels: lowest (<10%), low (10–30%), medium (30–50%), high (50–70%), and highest (>70%). The detailed levels, criteria, and associated surface landscape characteristics are shown in Table 2. The classification standards were derived from the Federal Geographic Data Committee (FGDC) and the United States Department of Agriculture (USDA) Forest Service guidelines on vegetation coverage grading, with slight modifications made to suit local realities.

#### 2.3.2. Trend Analysis

(1) Theil–Sen Median Trend Analysis and Mann–Kendall Test

The Theil–Sen Median [35] method is a robust non-parametric statistical method used for trend analysis [36]. This approach provides higher computational efficiency than the traditional least squares method and is insensitive to measurement errors and outliers, which makes it particularly suitable for long-term series analysis. The calculation formula is as follows [37]:

(2) $$\beta =median(\frac{X_{j}-X_{i}}{j-i})(2000\leq i<j\leq 2024)$$

where *β* represents the rate of FVC change, while *X_i_* and *X_j_* denote FVC values. When *β* > 0, it signifies an increasing trend, and when *β* < 0, it signifies a decreasing trend.

The Mann–Kendall test [38,39] is a non-parametric method for detecting trends in time series data. It does not require data to satisfy normal distribution and is resilient to outliers, making it ideal for testing the long-term trends [40]. The calculation formula is as follows [41]:

(3) $$S=\sum_{i=1}^{n-1}\sum_{j=i+1}^{n}sign(X_{j}-X_{i})$$

(4) $$\mathrm{var}(S)=\frac{n(n-1)(2n+5)}{18}$$

(5) $$Z=\left\{\begin{array}{c} \frac{s-1}{\sqrt{\mathrm{var}(S)}}\\ 0\\ \frac{s+1}{\sqrt{\mathrm{var}(S)}} \end{array}\right.\begin{array}{c} ,S>0\\ ,S=0\\ ,S<0 \end{array}$$

In the formula, var represents the variance function, and Z is the significance statistic. A significance level of 99% (Z > 2.58) was used to identify a significant trend in this study. The detailed grading standards are shown in Table 3.

(2) Hurst Index

The Hurst index, introduced by British hydrologist Hurst in 1951 [42], measures the long-term memory in a time series and how past information influences future trends. The calculation is based on the rescaled range analysis (R/S), expressed by the following formula:

(6) $$X(t)=\sum_{i=1}^{m}\left(\Delta X_{i}-\bar{\Delta X}(m)\right)$$

(7) R(m)=maxX(t)−minX(t)(1≤m≤n)

(8) $$S(m)={\left(\frac{1}{m}{\sum_{i=1}^{m}\left(\Delta X_{i}-\bar{\Delta X}(m)\right)}^{2}\right)}^{\frac{1}{2}}$$

(9) $$\frac{R(m)}{S(m)}=c\times m^{H}$$

Equation (6) computes the cumulative deviation, Equation (7) determines the range, Equation (8) calculates the standard deviation, and Equation (9) derives the H value through fitting. The H value ranges from [0, 1]. When H = 0.5, the time series is considered a random walk, unrelated to past trends. When H > 0.5, the time series exhibits long-term positive correlation, indicating the trend is likely to persist. Conversely, when H < 0.5, the time series shows long-term negative correlation.

Compared to complex models like random forests or deep learning, which require extensive computational resources and large datasets, the Hurst index offers a simple and efficient approach for analyzing large-scale, long-term series data. However, it may not be able to effectively capture trend changes when the data are highly volatile. To address its robustness, this study proposed an innovative approach by integrating Sen + M-K analysis with Hurst index to predict future vegetation trends. This combined method leverages the simplicity and long-memory properties of the Hurst index while enhancing trend detection robustness through Sen + M-K analysis, which is less sensitive to outliers. By identifying regions with increasing or decreasing trends and using a Hurst value of 0.5 as the threshold to classify future vegetation cover trends in Xinjiang into five levels, this approach can improve the accuracy. The detailed grading standards are shown in Table 4.

#### 2.3.3. Stability Analysis

The coefficient of variation (CV) quantifies the extent of variability relative to the mean of the variable [43]. This standardized measure of dispersion was selected because it can enable direct comparison of variability across variables with different units. Meanwhile, it provides a dimensionless relative measure that remains meaningful when the mean values differ substantially. These characteristics make CV more appropriate for our analysis than absolute measures of variability like standard deviation. A smaller CV indicates lower variability and greater stability [44]. The calculation formula is as follows:

(10) $$c=\frac{1}{d}\sqrt{\frac{\sum {\left(d_{i}-d\right)}^{2}}{n-1}}$$

In the formula, *d_i_* denotes the vegetation cover for the *i*-th year, and *d* is the mean. To assess vegetation cover stability in Xinjiang, the degree of variation is classified into five levels based on CV, as shown in Table 5.

#### 2.3.4. Correlation Analysis

Correlation analysis quantifies the strength of relationships between factors. In complex systems with multiple interacting variables, simple correlation coefficients may fail to reveal true relationships. The partial correlation method addresses this by isolating the effects of other variables, enabling a more focused analysis. In this study, the partial correlation method was applied to analyze the relationships between vegetation cover and two climatic factors—the annual mean temperature and the annual precipitation. The formulae are as follows:

(11) $$R_{xy}=\frac{\sum_{i=1}^{n}\left(x_{i}-\bar{x})(y_{i}-\bar{y}\right)}{\sqrt{\sum_{i=1}^{n}{\left(x_{i}-\bar{x}\right)}^{2}}\sqrt{\sum_{i=1}^{n}{\left(y_{i}-\bar{y}\right)}^{2}}}$$

(12) $$R_{xy\cdot z}=\frac{R_{xy}-R_{xz}\cdot R_{yz}}{\sqrt{\left(1-{R_{xz}}^{2})(1-{R_{yz}}^{2}\right)}}$$

#### 2.3.5. Geographical Detector

The GeoDetector [45], developed by Professor Wang Jingfeng and his team, is a set of statistical methods for detecting spatial heterogeneity and identifying driving factors [46]. This approach was adopted because it effectively quantifies the spatial stratified heterogeneity of variables and provides a robust measure (*q*-statistic) to assess the influence of potential driving factors, including both numerical and categorical variables. It avoids linear assumptions, making it suitable for detecting nonlinear relationships and interactions between factors. It has been widely validated in geographical and environmental studies, ensuring methodological reliability. It includes four main components:

(1) Factor detector: It assesses the spatial heterogeneity of an attribute *Y* and quantifies how much a factor *X* explains this heterogeneity. The degree is quantified by *q*-value, calculated using the following formula:

(13) $$q=1-\frac{\sum_{h=1}^{L}N_{h}{\sigma_{h}}^{2}}{N\sigma^{2}}=1-\frac{SSW}{SST}$$

The *q*-value ranges from 0 to 1, with higher values indicating stronger spatial heterogeneity of *Y*.

(2) Interaction detector: It evaluates whether the combined effects of *X*_1_ and *X*_2_ enhance, weaken, or independently contribute to their explanatory power for the dependent variable *Y* by comparing *q*(*X*_1_), *q*(*X*_2_) and *q*(*X*_1_∩*X*_2_). The detailed grading standards are shown in Table 6.

(3) Risk detector: It is used to assess whether significant differences exist in mean attribute values between two subregions using the t-statistic. The formula is as follows:

(14) $$t_{\bar{y}h=1}-{\bar{y}}_{h=1}=\frac{{\bar{Y}}_{h=1}-{\bar{Y}}_{h=2}}{{\left[\frac{Var({\bar{Y}}_{h=1})}{n_{h=1}}+\frac{Var({\bar{Y}}_{h=2})}{n_{h=2}}\right]}^{1/2}}$$

The null hypothesis (*H*_0_) states

(15) $${\bar{Y}}_{h=1}={\bar{Y}}_{h=2}$$

If *H*_0_ is rejected at a confidence level *α*, it suggests the existence of significant differences in the mean attribute values between the two subregions.

(4) Ecological detector: It is used to compare whether two factors have significantly different effects on the spatial distribution of *Y*. The comparison is evaluated using the F-statistic, calculated as follows:

(16) $$F=\frac{N_{X1}(N_{X2}-1)SSW_{X1}}{N_{X2}(N_{X1}-1)SSW_{X2}}$$

The *H*_0_ assumes

(17) $${SSW}_{X1}={SSW}_{X2}$$

## 3. Results

### 3.1. Spatiotemporal Changes and Distribution of Vegetation Cover

#### 3.1.1. Spatial Distribution Characteristics

**Figure 4:**
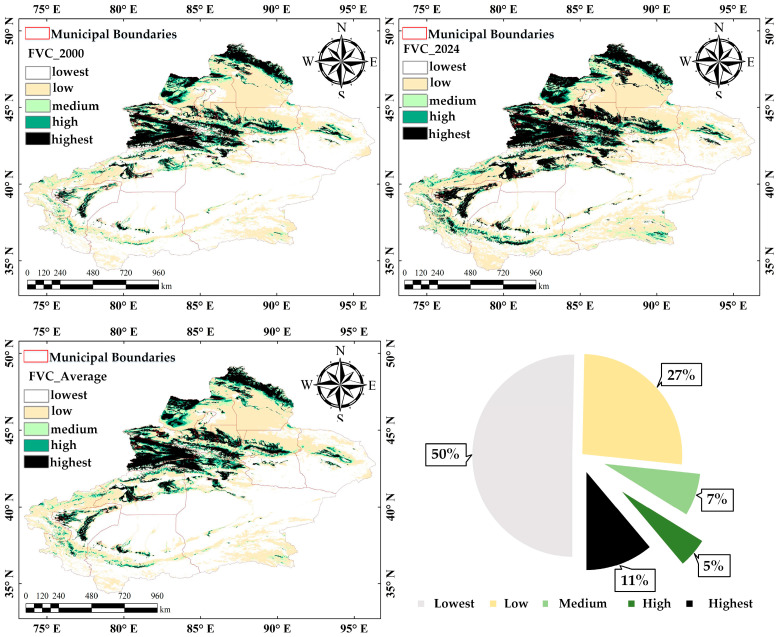
Spatial distribution of vegetation cover at different levels.

By comparing the spatial distribution maps of vegetation cover in Xinjiang for the years 2000, 2024, and the average for 2000–2024 (Figure 4), notable regional differences and gradient patterns are evident. Areas with high and highest vegetation cover are widely distributed in northern Xinjiang, including the northern Tianshan Mountains, Altai Mountains, and Ili River Valley. These regions receive higher precipitation and abundant snowmelt water resources, providing favorable conditions for vegetation growth. In contrast, areas with low and lowest vegetation cover, such as the Taklamakan Desert, suffer from extreme aridity and low precipitation. These areas remain sparsely vegetated over extended periods. Overall, the vegetation cover in Xinjiang follows a clear gradient, with the north having significantly higher cover due to favorable climate and water resources.

#### 3.1.2. Temporal Variation Characteristics

**Figure 5:**
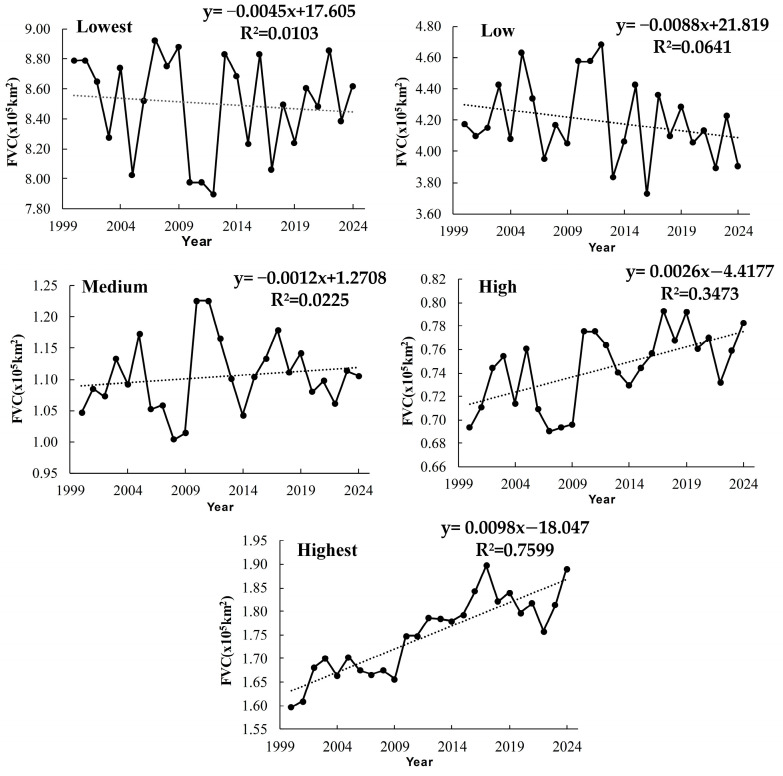
Changes in Xinjiang vegetation cover levels from 2000 to 2024.

By comparing the changes in the area of different levels of vegetation cover in Xinjiang from 2000 to 2024 (Table 7 and Figure 5), it becomes evident that the overall vegetation condition in Xinjiang has shown gradual improvement. However, spatiotemporal variations and instability persist.

The area with the lowest vegetation cover decreased slightly from 53.91% to 52.89%. However, significant interannual fluctuations were observed, highlighting the ecological fragility of these regions. A notable reduction in bare land occurred between 2009 and 2012, likely due to rapidly increased precipitation, which temporarily supported vegetation growth in areas like oasis edges and grasslands. However, bare land expanded again in 2013 and 2024, possibly due to drought or human activities causing vegetation degradation. The decrease in low vegetation cover suggests some regions may have potential for ecological recovery under suitable conditions. However, the effectiveness may be undermined by natural disasters or extreme climatic events.

The area of medium vegetation cover showed a slight overall increase, serving as a dynamic ecological buffer zone. Its fluctuations reflect the interplay between vegetation improvement and degradation, influenced by both climate change and human activities. As shown in Figure 5, the reduction in lowest vegetation cover coincided with an increase in low and medium vegetation cover between 2009 and 2012, highlighting the success of desertification control efforts during that period.

Notably, areas with high and highest vegetation cover have continued to expand, reflecting an overall improvement in vegetation quality. The area of high vegetation cover grew from 4.25% in 2000 to 4.80% in 2024. The area of highest vegetation cover saw a more significant increase, rising from 9.80% in 2000 to 11.60% in 2024, a growth of 1.80%. The most rapid growth occurred between 2015 and 2017, driven by large-scale vegetation restoration projects, the expansion of oasis agriculture, and improved irrigation systems. In these regions, climate improvements and ecological restoration measures work together to create a positive feedback loop that supports vegetation growth.

Overall, the evolution of vegetation cover levels in Xinjiang from 2000 to 2024 demonstrates encouraging signs of regional ecological enhancement. However, the enhancement remains unstable. Therefore, future ecological management and planning must rely on robust scientific monitoring and comprehensive data analysis. Targeted and refined strategies should be implemented to improve stability and resilience.

#### 3.1.3. Comparison Verification

Verifying the accuracy of the FVC inversion results is of great significance to affirming the value of this study. However, due to the lack of large-scale ground-based measured data in Xinjiang, it is difficult to carry out traditional direct verification based on sampling. Therefore, this study adopted a verification scheme of spatial consistency comparison with the GLASS global FVC product, which adopts a strict training sample screening mechanism and model optimization strategy and can provide a reliable physical basis. However, due to the extensive data gaps in the GLASS product over Xinjiang, this study could only conduct comparative validation in locally available coverage areas. Among these, the Ili River Valley serves as a typical ecological transition zone in Xinjiang, exhibiting vegetation characteristics of both northern and southern Xinjiang. It hosts six provincial-level nature reserves, and its ecosystem holds significant representativeness in the regional ecological landscape of Xinjiang. Based on this, this study selected the central area of the Ili River Valley as the validation zone. This study selected a 6-year period for verification.

**Figure 6:**
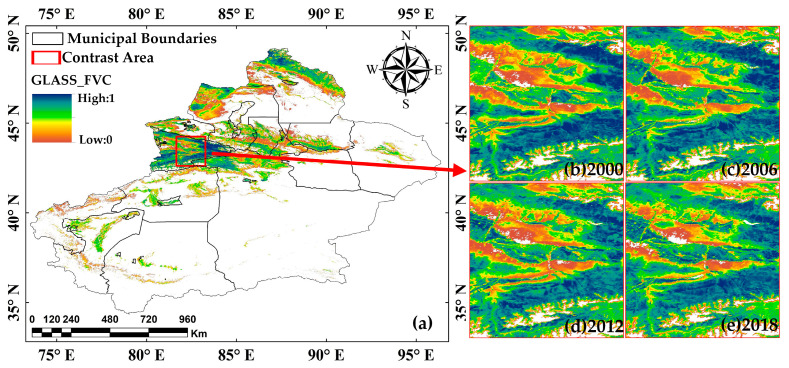
Geographic location (**a**) and GLASS_FVC data of the validation zone (**b**–**e**).

The analytical procedure consisted of three steps: (1) Obtain and merge Xinjiang GLASS FVC products from June to August in 2000, 2006, 2012, and 2018. (Figure 6). (2) Preprocessing (including cropping, projection, spatial matching, resampling, normalization). (3) Perform quantitative comparative analysis using pixels with valid values in both. This study employed five quantitative metrics to assess the similarity: Mean Absolute Error (MAE), Root Mean Square Error (RMSE), Coefficient of Determination (R²), Structural Similarity Index (SSIM), and Peak Signal-to-Noise Ratio (PSNR). MAE and RMSE values were converted into similarity measures using min-max normalization for consistent comparison. The evaluation results are presented in Table 8.

The multi-metric validation results in Table 8 demonstrate the high credibility of the FVC inversion results, with consistently stable metrics during 2006–2018 (MAE ≈ 0.9976, RMSE ≈ 0.951, SSIM > 0.98, PSNR > 26), indicating excellent temporal consistency and robustness. The SSIM and PSNR values confirm that the spatial patterns of retrieved FVC show strong agreement with reference data, meeting the accuracy requirements for regional ecological monitoring. However, the notably lower metric values in 2000 may be attributed to limitations in early-period remote sensing-data quality. Further studies incorporating ground-based measurements are recommended to investigate the causes of these discrepancies in the 2000 results.

### 3.2. Analysis of Vegetation Cover Change Trends and Stability

#### 3.2.1. Trend Analysis of Changes

**Figure 7:**
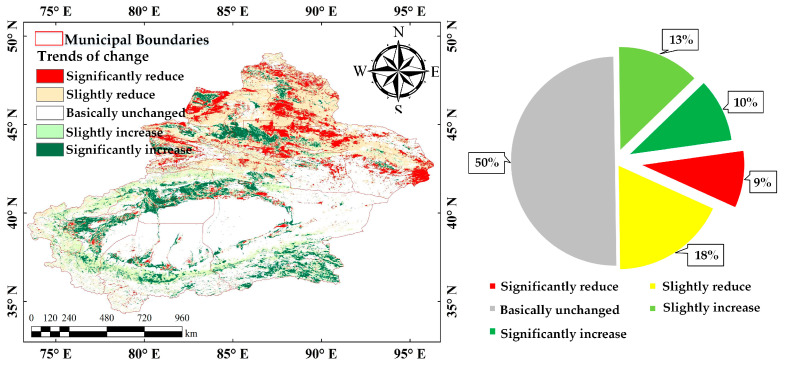
Trend analysis of vegetation cover changes from 2000 to 2024.

Based on Theil–Sen Median trend analysis and the M-K test, the vegetation cover changes in Xinjiang from 2000 to 2024 show significant spatial variation (Figure 7). Areas with essentially unchanged vegetation cover constitute the largest proportion, accounting for 50%. These areas are extensively distributed across deserts and arid grasslands, where extreme climatic conditions result in consistent low cover. Areas with slight improvement, representing 13%, are mainly found in oasis zones and the Tianshan Mountain slopes, and some arid and semi-arid grasslands. Areas with a significant increase in vegetation cover represent 10% and are mainly located in the humid northern regions. These regions benefit from relatively favorable climatic conditions, which, when supported by relevant policies, promote rapid vegetation restoration.

Areas with a slight reduction in vegetation cover represent 18% and are primarily located along the edges of oases, transitional zones of northern Xinjiang grasslands. Areas with a significant reduction constitute 9% and are primarily found in the northern slope of the Tianshan Mountains and certain agricultural development zones in oasis economic areas, likely due to urban expansion, overuse of agricultural land, and resource extraction.

Overall, vegetation cover changes in Xinjiang reflect both improvement and degradation. While ecological restoration projects have achieved significant progress, certain localized areas remain at risk of degradation. So, future ecological management should focus on refined, targeted restoration strategies for the fragile regions.

#### 3.2.2. Stability Analysis of Changes

**Figure 8:**
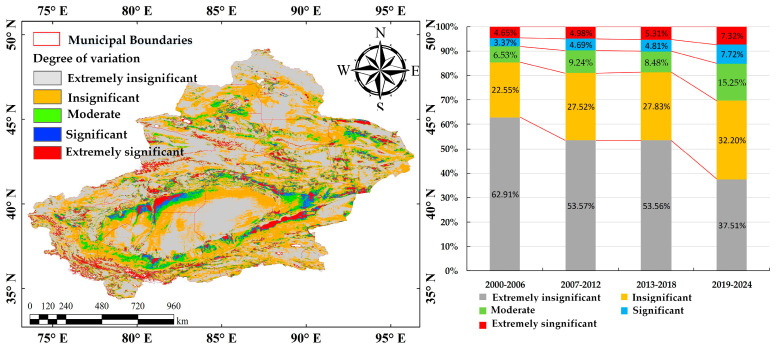
Changes in the stability of vegetation cover from 2000 to 2024.

The CV of vegetation cover was analyzed for four periods between 2000 and 2024 (Table 9), with the spatial distribution of mean CV values at different vegetation cover levels (Figure 8). The results reveal significant temporal variation in CV and notable changes in the proportion of areas with different stability levels. Moreover, the stability of vegetation cover in Xinjiang has shown a declining trend. Specifically, the proportion of areas with lower CV values (CV 0–0.25) has decreased from 85.46% to 69.71%, while areas with higher CV values (CV > 0.35) have increased from 8.02% to 15.04%. The result indicates increased vegetation fluctuations and reduced stability in certain regions. To achieve sustainable development of regional ecosystems, efforts should focus on consolidating the ecological benefits of highly stable regions while enhancing ecological restoration in high-fluctuation areas.

In order to deeply analyze the regional differentiation laws of the spatiotemporal variation characteristics of vegetation coverage, this study calculated the coefficient of variation (CV) of FVC from 2000 to 2024 based on the above-mentioned five ecological sub-regions in Xinjiang. The results show the following (Table 10): (1) The Altai mountains–western Junggar mountain forest and grassland ecological region (I) exhibits the highest vegetation stability, with 88.93% of areas showing insignificant CV (CV < 0.25), attributable to its intact forest ecosystems and favorable hydrothermal conditions. (2) Zone IV (Tarim Basin–Eastern Xinjiang Desert Ecological Zone) and Zone V (Pamir-Kunlun Mountains–Altun Mountains Alpine Desert Steppe Ecological Zone) both exhibit higher proportions of areas with “Moderate” to “Significant” fluctuations, especially in Zone V, where the “Extremely significant” category reaches 12.77%, highlighting the instability of vegetation cover in these desert and mountainous areas. These results indicate considerable regional variation in vegetation stability, which underscores the need for targeted ecological restoration efforts based on specific regional characteristics.

#### 3.2.3. Future Development Trend Analysis

**Figure 9:**
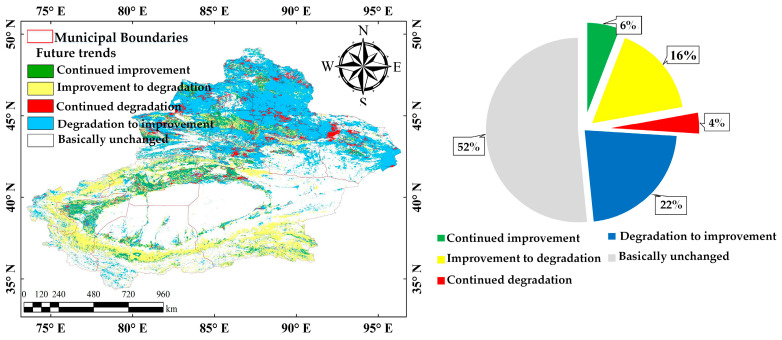
Future development trend analysis of vegetation cover in Xinjiang.

Based on the Hurst index and Sen–MK trend analysis, the potential future development trends of Xinjiang vegetation cover show significant regional variation (Figure 9).

The results show that areas with unchanged vegetation cover account for 52%, the largest proportion, mainly in deserts and parts of the desert grasslands. Due to harsh natural conditions, vegetation in these areas may remain stable with low coverage over an extended period.

Areas with continued improvement account for 6%, mainly in the northern Tianshan slopes, Ili River Valley, and desert-edge oases, benefiting from ecological restoration projects. The improvement-to-degradation areas make up 16% and mainly found on oasis edges and northern Xinjiang’s transitional zones. This suggests that the ecological restoration effects in these areas are reversible and proactive monitoring and management measures should be adopted to mitigate the risk of degradation.

Areas with continued degradation account for 4%, mainly in intensively developed oasis regions and ecologically fragile zones. These areas often face significant ecological pressure resulting from urban expansion, agricultural development, and excessive resource utilization. Though small in proportion, the degradation could impact local ecosystems. So, enhanced management is necessary to regulate human activities to curb ecological degradation. The degradation-to-improvement areas make up 22% and are concentrated in the Tarim River Basin, oasis edges, and parts of the arid zones in northern Xinjiang, showing obvious recovery potential.

Overall, the potential future evolution of vegetation cover in Xinjiang follows a general trend of being “predominantly stable, locally improving and degrading.” In the future, location-specific and differentiated management measures should be implemented to enhance dynamic monitoring and risk-warning systems.

### 3.3. Analysis of Driving Factors

#### 3.3.1. Spatial Scale Selection and Optimal Discretization

Determining an appropriate analysis scale is critical [47,48] before assessing the influence of various driving factors. Scale selection directly affects the representation of spatial data characteristics. At larger scales, the assessment of ecological factors may be influenced by dominant land ecological elements within units and may obscure differences among distinct ecological subsystems. Conversely, assessments conducted at smaller scales often lack generalizability for large-scale ecological applications.

**Figure 10:**
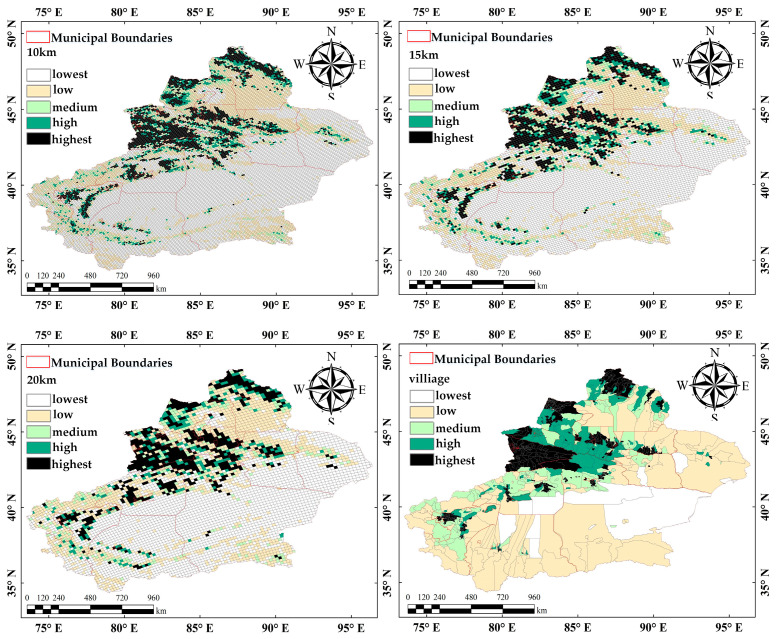
Interpolation of Xinjiang FVC at different spatial scales.

**Figure 11:**
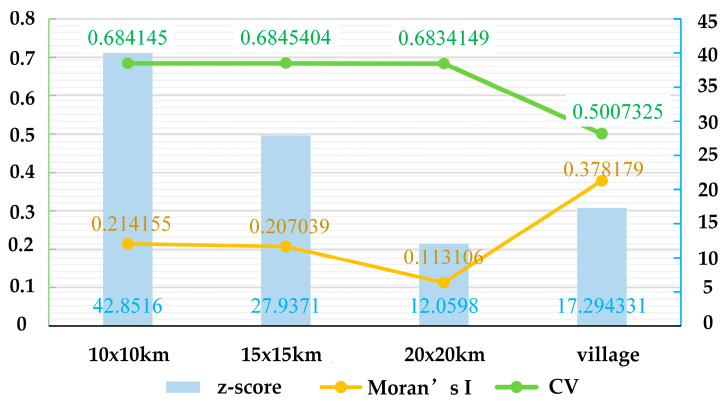
Moran’s I and coefficient of variation at different scales.

In this study, gridded sampling was used to construct two types of spatial units across four scales: plot-based (10 × 10 km, 15 × 15 km, 20 × 20 km) and township-based unit (Figure 10). The plot-based scales were selected to capture varying levels of ecological detail, enabling the examination of fine- to broad-scale spatial patterns. The township-based unit was chosen to align with administrative boundaries, facilitating data integration and policy-relevant applications. This multi-scale approach ensures a comprehensive analysis of spatial patterns and processes across different resolutions. Subsequently, a comparative analysis was conducted on the spatial autocorrelation and CV of remote sensing parameters at different scales within the study area (Figure 11) to analyze their response patterns to scale variations. Spatial autocorrelation quantifies the spatial distribution patterns, and CV reflects the richness of information at a given scale.

Moran’s I values for FVC at the four scales were 0.214, 0.207, 0.113, and 0.378, with higher values reflecting stronger spatial clustering. Additionally, the Z-scores for all scales exceeded 1.96, meeting the significance level. However, as the spatial extent increased, Moran’s I for the 20 × 20 km scale dropped significantly, indicating weaker spatial clustering strength. Furthermore, the CV at the township-based scale was markedly lower, suggesting less information. But the 15 × 15 km grid scale exhibited both a Moran’s I value comparable to other scales and a relatively higher CV. Therefore, this study selected the 15 × 15 km scale as the analysis scale to balance the need for revealing the spatial-clustering characteristics of FVC and capturing spatial variability.

**Figure 12:**
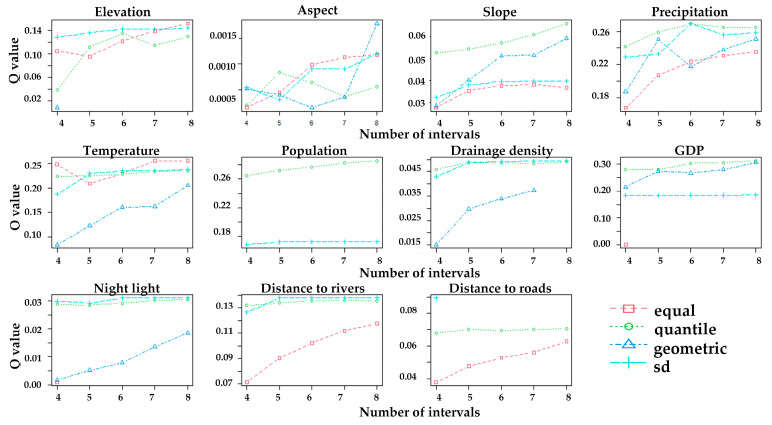
Optimal discretization of driving factors.

The driving factors include both continuous and discrete variables. Four discrete variables—landform, soil type, vegetation type and land use type—were reassigned according to the original classification and then included in the GeoDetector analysis. Continuous variables need to be discretized. Four discretization methods—equal interval, quantile classification, geometric interval, and standard deviation—were applied to determine the optimal discretization for each factor. This approach helps identify the underlying mechanisms between vegetation cover and its driving factors [49]. The final optimal discretization results are shown on Figure 12.

#### 3.3.2. Results of GeoDetector Analysis

**Figure 13:**
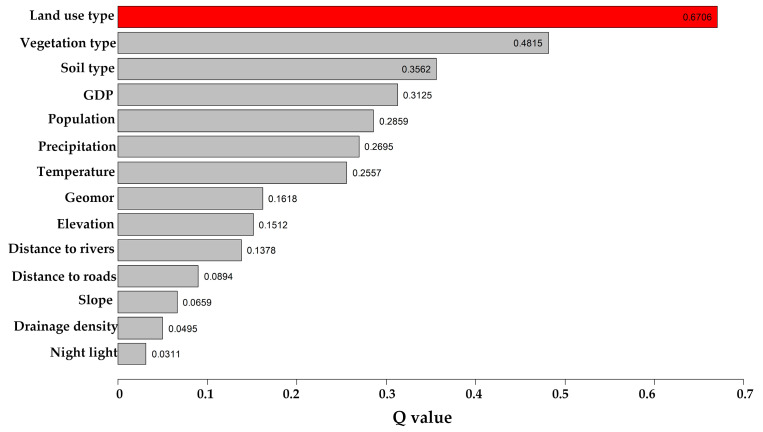
The *q*-values of the driving factors (Red represents the factor with the largest q value).

(1) The factor detector was used to evaluate the influence of multiple driving factors on vegetation cover, as illustrated in Figure 13. The ranking of *q*-values is as follows: land use type (0.6706) > vegetation type (0.4815) > soil type (0.3562) > GDP (0.3125) > population (0.2859) > precipitation (0.2695) > temperature (0.2557) > geomorphology (0.1618) > elevation (0.1512) > distance to rivers (0.1378) > distance to roads (0.0894) > slope (0.0659) > drainage density (0.0495) > night light (0.0311).

Among these factors, land use type is the dominant factor. Vegetation type is the most influential natural factor. Precipitation and temperature are key climatic driving factors. Topographic factors, such as geomorphology and elevation, exert relatively weaker impacts, while aspect (*q* = 0.0018) has a negligible influence. Among human factors, night light and distance to roads have relatively weak effects.

Overall, changes in vegetation cover in Xinjiang are driven by a combination of natural and human factors. Future ecological management should focus on land use planning and vegetation management, while considering climatic and topographic conditions to implement targeted strategies for ecosystem sustainability.

**Figure 14:**
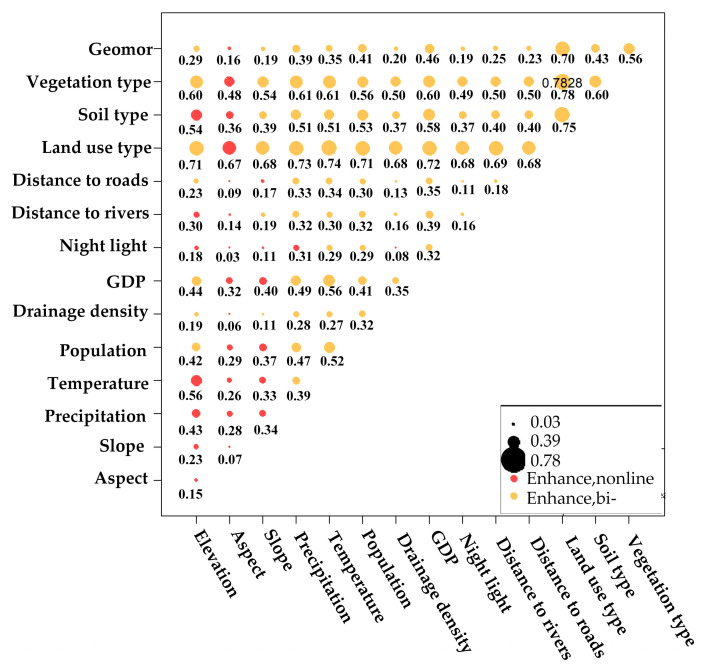
Interaction detection of driving factors.

(2) The interaction detector assessed how factor interactions influence the explanatory power of vegetation cover in Xinjiang. The results (Figure 14) reveal that factor interactions fall into two categories: nonlinear enhancement and linear enhancement (totally 105 pairs). In all cases, the combined influence of two factors offered greater explanatory power for FVC than individual factors, highlighting the significance of factor coupling in shaping the spatiotemporal differentiation of FVC.

Among these interactions, linear enhancement relationships (77 pairs) outnumber nonlinear enhancement relationships (28 pairs). The interaction between land use type and vegetation type offers the strongest explanatory power, with a linear enhancement *q*-value of 0.7828. This is followed by the interaction between land use type and soil type, with a *q*-value of 0.7495. Conversely, the interaction between aspect and night light has the weakest explanatory power, demonstrating a nonlinear enhancement with a *q*-value of 0.0340. Although the individual *q*-values for aspect and drainage density are among the lowest, their interaction still makes an improvement in explanatory power. These findings highlight the intricate interaction mechanisms between natural and human factors shaping vegetation cover in Xinjiang.

**Figure 15:**
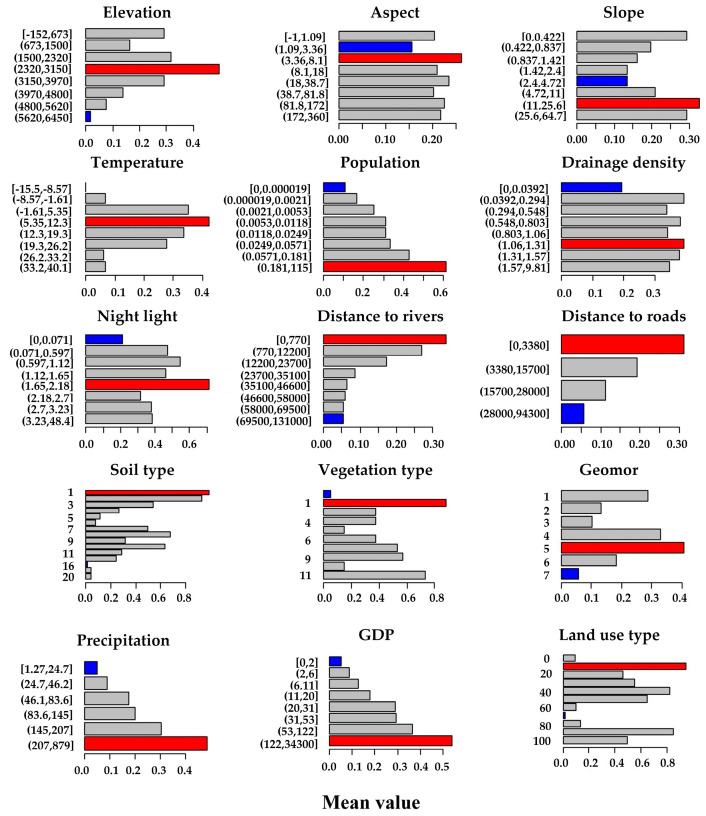
Risk detection of driving factors.

(3) The risk detector was used to quantitatively assess the mean differences in FVC under different driving factors, with the results shown in Figure 15. This figure presents the risk matrix of FVC across various driving factors in Xinjiang. By comparing the mean attributes across sub-regions, optimal ranges for factors influencing vegetation growth can be identified.

The optimal elevation range for vegetation cover in Xinjiang is between 2320 and 3150 m, mainly in the mid-to-high mountain regions of the Altai and Tianshan ranges. These regions have a humid climate, providing favorable conditions for vegetation growth. In contrast, desert lowlands and high-altitude cold regions exhibit much lower vegetation cover due to extreme environmental conditions. FVC attains higher values in regions with slopes between 11° and 25.6°, where moderate slopes facilitate soil drainage and nutrient accumulation. Climatically, vegetation cover peaks in regions with higher precipitation and annual mean temperatures between 5.35 °C and 12.3 °C, where optimal water and heat conditions interact synergistically to promote vegetation growth.

In terms of anthropogenic factors, vegetation cover shows a positive correlation with population density and GDP, which are closely tied to Xinjiang’s distinctive desert geography. Population and economic activities are primarily concentrated in oasis and urban areas, where better water resources and agricultural infrastructure support vegetation growth. FVC is higher near rivers and roads, with rivers providing stable irrigation and roads supporting oasis agriculture and ecological management. When night light intensity falls in the range of 1.65–2.18, vegetation cover peaks. In contrast, areas with excessively high night light intensity, such as urban cores, tend to have lower FVC due to the prevalence of impervious surfaces.

Identifying the optimal ranges of these driving factors not only uncovers the intricate mechanisms by which natural and anthropogenic factors shape vegetation growth but also offers a scientific basis for vegetation restoration and resource optimization in Xinjiang.

**Figure 16:**
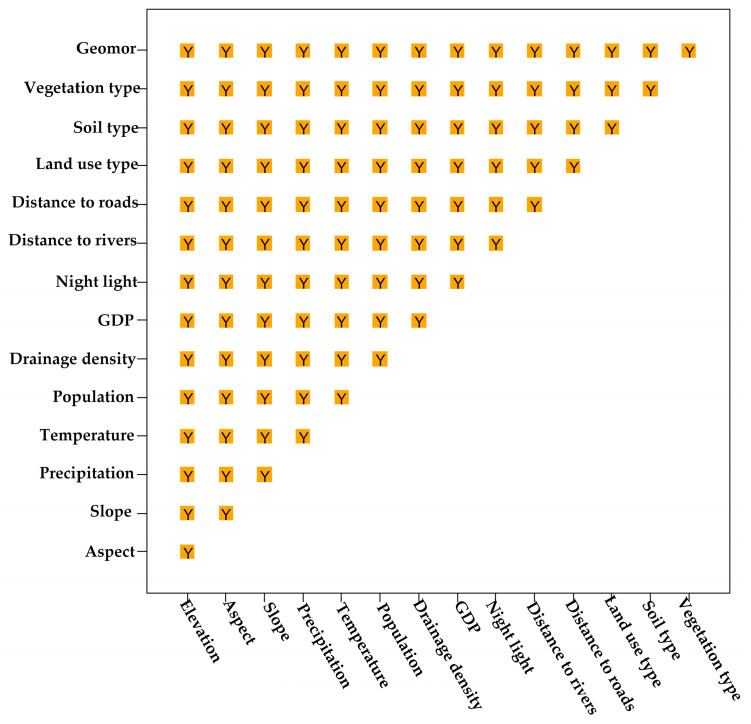
Ecological detection of driving factors.

(4) The ecological detector was used to assess whether any two factors exhibit significantly different effects on the spatial distribution of the FVC. The results (Figure 16) show significant differences in the effects of all factor pairs. This finding indicates that the 15 selected factors exhibit no redundancy, as each factor exerts a unique and significant influence on FVC to varying degrees, which further confirms the validity and scientific relevance of the selected factors.

#### 3.3.3. Analysis of Driving Factors Based on Ecological Sub-Regions

To elucidate the spatial heterogeneity of vegetation coverage drivers, this section employed factor detector and interaction detector analyses within Xinjiang’s five ecological sub-regions, enabling a comparative assessment of driving mechanisms.

**Figure 17:**
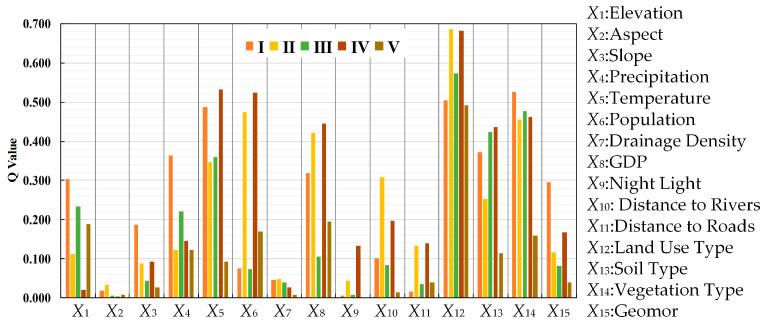
Factor detection in the five sub-regions in XinJiang.

The results of the factor detection in the five sub-regions are shown in Figure 17. The results demonstrate significant spatial heterogeneity in dominant driving factors across ecological zones. In mountainous forest ecosystems (I), climatic factors (precipitation and temperature) dominate vegetation coverage through hydrothermal regulation of boreal forests, showing strong interactions with topographic variables. The Tianshan grassland region (III) exhibits pronounced synergistic effects among land use type, vegetation type, and soil type, reflecting grazing–vegetation feedback mechanisms. Desert and semi-desert regions (II, IV, V) display predominant anthropogenic influences from land use and socioeconomic factors (population, GDP), indicating intensive human modification. The results show that the driving mechanism of vegetation coverage in Xinjiang has obvious spatial differentiation characteristics. The mountain forest area shows a stable regulation mode dominated by natural factors. The grassland cover belt shows the effects of both natural and human factors, and the impact of human activities is significantly enhanced. The desert area forms an irreversible pattern dominated by human activities. This significant difference between sub-regions shows that the interactive complexity of ecological location and environmental coercion is crucial to the evolution of vegetation cover, and it is necessary to formulate topological ecological management measures from a multi-dimensional comprehensive consideration.

The factor detector analysis (Figure 17) revealed that land use type exhibited the highest explanatory power for vegetation coverage changes across all ecological sub-regions in Xinjiang (with the greatest individual *q*-values). To further elucidate the synergistic mechanisms of driving factors in different ecological zones, this study focused on analyzing the interaction effects between land use type and other driving factors. Results demonstrated that these interactions significantly enhanced explanatory power in all sub-regions.

By extracting the top three most influential factor interactions (Table 11), we identified key regional characteristics: (1) In the Altai–western Junggar Mountain Forest and Grassland Ecological Region (Zone I), the land-use–temperature interaction dominated, reflecting the high sensitivity of boreal forest ecosystems to local thermal variations. (2) Both the Junggar Basin Desert Ecological Zone (Zone II) and Tarim Basin–Eastern Xinjiang Desert Ecological Zone (Zone IV) showed the strongest land-use–population density interactions, indicating significant anthropogenic impacts on desert vegetation through grazing, agricultural expansion, conservation measures, and so on. (3) The Tianshan Mountain Forest and Grassland Ecological Region (Zone III) exhibited the most pronounced land-use–vegetation type interaction, demonstrating differential responses of vegetation types along elevation gradients to ecological management. (4) The Pamir-Kunlun-Altun Mountains alpine desert steppe ecological zone (Zone V) was characterized by dominant land-use–elevation interactions, highlighting the strong dependence of alpine ecosystems on topographic factors. The altitude gradient indirectly affects vegetation growth by regulating the water and heat redistribution process.

It is noteworthy that land use, vegetation type, temperature, population density, and soil type all demonstrate strong explanatory power in the interaction effects across different regions, which fully confirms the universal significance of these factors as key drivers of vegetation cover changes in arid areas. The differences in explanatory power exhibited by these factors across various ecological zones also reveal significant spatial heterogeneity in the driving mechanisms of vegetation cover changes. The study holds important ecological management value: (1) The specific combination of factors in each ecological zone can serve as indicative indicators for ecosystem health assessment. (2) The quantified interaction intensities provide operational threshold references for formulating regionally differentiated conservation strategies. (3) The revealed spatial patterns offer scientific support for prioritizing areas in ecological restoration projects. These mechanistic insights significantly enhance our ability to predict arid region vegetation responses to both natural environmental changes and human activities.

### 3.4. Partial Correlation Analysis of Vegetation Cover and Climatic Factors

**Figure 18:**
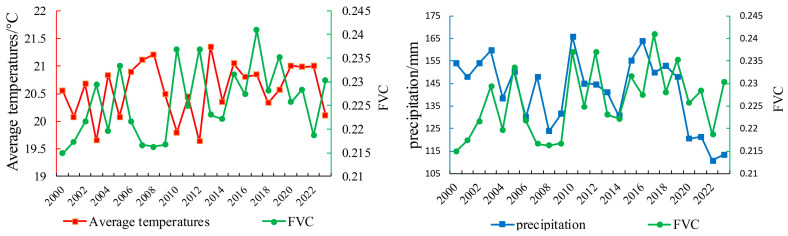
Comparison of annual mean temperature, precipitation, and FVC from 2000 to 2024.

**Figure 19:**
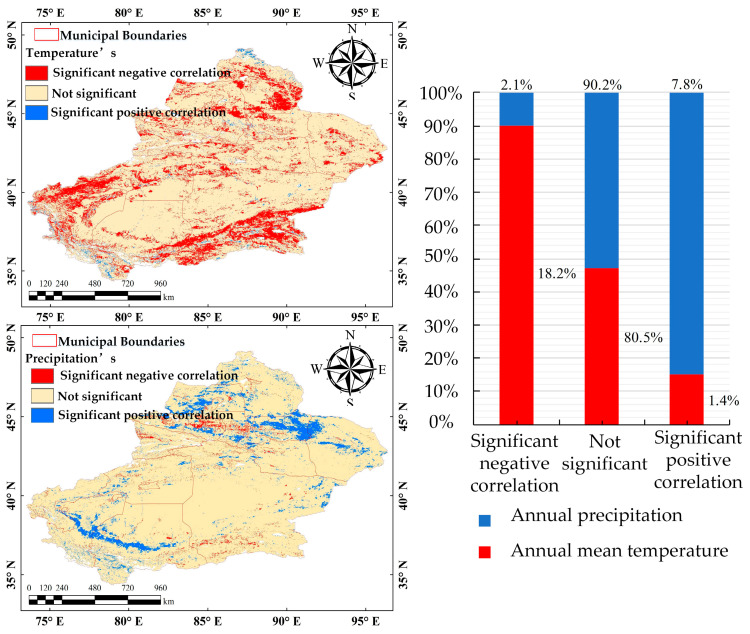
Partial correlation of FVC with annual mean temperature (**left**) and annual precipitation (**right**).

Climatic factors have long been a critical component of ecological research. To further explore the impact of climatic factors, this study analyzed annual mean temperature and precipitation in XinJiang and compared them with corresponding annual mean FVC values (Figure 18). Additionally, partial correlation coefficients were used to measure the independent effects of temperature and precipitation on FVC (Figure 19).

Temperature and precipitation in Xinjiang show significant geographic variation. High-altitude mountainous regions are cooler, while basin areas and oases in southern Xinjiang are warmer. And precipitation follows a distinct north-high, south-low gradient.

From 2000 to 2023, Xinjiang’s annual mean temperature and precipitation showed irregular fluctuations, with complex interactions affecting FVC stability and trends. Precipitation has a more direct impact on FVC. When it increases (e.g., 2001–2003 and 2009–2010), FVC rises. In years with sufficient precipitation (e.g., 2010 and 2017), FVC typically remains high. But when precipitation decreases (e.g., 2003–2004, 2005–2006, and 2019–2022), FVC drops significantly, emphasizing the importance of adequate precipitation for vegetation growth.

The impact of rising temperatures on FVC is more complex. Higher temperatures can extend the growing season, boosting FVC. But without enough precipitation, increased heat can lead to higher evaporation, causing soil moisture shortages and suppressing vegetation growth. This interaction is especially evident when temperatures rise while precipitation declines (e.g., 2003–2004 and 2005–2008).

A significance test (*p* > 0.05) was conducted on the partial correlation analysis results (Figure 19). The regions where FVC in Xinjiang is significantly positively correlated with annual mean temperature account for just 1%. These regions mainly are localized in high-altitude mountainous areas where low temperature limit vegetation growth, and a moderate increase in temperature will enhance FVC. In contrast, 18% of areas show a significant negative correlation, mainly in southern Xinjiang and around the edges of the Taklamakan Desert. In these areas, excessively high temperatures may exacerbate evaporation and hinder vegetation growth. Consequently, annual mean temperature has an overall negative impact on FVC in Xinjiang [50], particularly in high-temperature arid regions.

For annual precipitation, 8% of areas show a significant positive correlation with FVC, mainly in humid regions in northern Xinjiang and desert-edge oases in southern Xinjiang. Conversely, only 2% of areas show a negative correlation and are scattered along the desert fringes of the Junggar Basin and the northern slopes of the Tianshan Mountains. Overall, annual precipitation has a positive impact on FVC in Xinjiang, which further confirms the relationship between FVC and the trends of temperature and precipitation shown in Figure 18.

These findings reveal the complex interaction between climatic factors and FVC. Future efforts should focus on optimizing water resource management to mitigate stress from high temperature to support vegetation growth.

## 4. Discussion

Xinjiang, situated in the northwest frontier of China, serves as a critical ecological security barrier. Changes in vegetation dynamics in this region are important for national ecological security. This study reveals that areas with high vegetation coverage are predominantly concentrated in northern Xinjiang, including the Tianshan Mountains, Altai Mountains, and Ili River Valley, which aligns with the findings of Li et al. [51]. The proportion of high vegetation coverage areas in Xinjiang increased by 2.35%, while low vegetation coverage areas decreased by 2.71%, indicating a significant upward trend in the Fractional Vegetation Cover (FVC). The result is consistent with the observations reported by Zhao et al. [52].

To explore the mechanisms by which various driving factors influence vegetation dynamics in Xinjiang, this study analyzed 15 factors. The results indicate that among the natural factors, vegetation and soil type exert the most significant influence. The most suitable soil type for vegetation growth was identified as leached soil. The leached soil is rich in clay and natural fertility, which promotes optimal vegetation development. And the fact further validates the reliability of the factor analysis.

The next most influential factors are the two climatic variables, annual precipitation and annual mean temperature, contributing 27.0% and 25.6%, respectively. Precipitation directly determines the availability of water resources, while the annual mean temperature regulates the length of the growing season, growth rates, and water evaporation. In arid regions, adequate precipitation can greatly enhance vegetation growth, aligning with the findings of Wu et al. [53]. However, temperature increases have dual effects: moderate warming can extend growing season, but excessive heat, in the absence of sufficient precipitation, intensifies evaporation and reduces vegetation cover.

Anthropogenic factors also influence vegetation cover through various pathways, with the land use type being the most significant, aligning with the result of Zhao et al. [49]. The land use type directly reflects the disturbances and alterations caused by human activities, such as agricultural practices, urbanization, and resource extraction. For example, urban expansion often leads to the degradation of grasslands and forests. In addition to the land use type, GDP and population density demonstrate a considerable positive correlation with the FVC. In Xinjiang, areas with higher population density and GDP are typically concentrated in oasis regions, which benefit from relatively abundant water resources and favorable irrigation conditions. In contrast, sparsely populated desert and remote mountainous areas, with limited resources and infrastructure, tend to have lower vegetation cover.

Meanwhile, the significant increase in FVC in Xinjiang is closely tied to multiple ecological restoration projects, especially the Three-North Shelterbelt Project launched in 1978. Over 46 years of continuous construction, this project has formed a shelterbelt ecosystem dominated by arbor forests, effectively mitigating wind-blown sand, conserving soil and water resources, and promoting agricultural and pastoral development. Windbreak and desertification control initiatives around the Junger Basin closely match the notable greening areas identified through the Sen + MK trend analysis in this study. As the sixth phase of the Three-North Shelterbelt Project progresses (2021–2030) and the forest chief system is comprehensively implemented, Xinjiang’s shelterbelt construction enters a new stage of development. Future research can further integrate high-resolution remote sensing data to quantify the impact of ecological projects on FVC, offering scientific evidence for improving regional ecological restoration policies.

To elucidate the spatial heterogeneity of vegetation coverage drivers, this section employed factor detector and interaction detector analyses within Xinjiang’s five ecological sub-regions, enabling a comparative assessment of driving mechanisms. Land use type consistently emerges as the most influential factor in the five sub-regions, with its interactions with temperature, population density, and elevation showing particularly strong regional-specific effects (*q*-values 0.56–0.79). Meanwhile, the differences in explanatory power exhibited by these factors across sub-regions also reveal significant spatial heterogeneity in the driving mechanisms of vegetation cover changes. Such mechanistic understanding substantially improves predictive capacity regarding vegetation dynamics under changing environmental conditions and anthropogenic pressures, enabling more effective and evidence-based decision-making for sustainable dryland management.

This study explored the factors influencing vegetation cover in Xinjiang from multiple dimensions. However, some limitations remained. First, factors such as groundwater depth, agricultural irrigation, and the nature reserves were not considered. Second, due to the spatial and temporal resolution constraints of the data, the dynamic changes in certain driving factors and their actual impact on FVC might not have been comprehensively captured. Additionally, this study primarily relied on remote sensing and statistical models and lacks sufficient field survey data for validation. Future research should address these limitations by expanding and refining the analysis. For instance, groundwater depth and irrigation data could be integrated using hydrological models. The impact of nature reserves could be assessed by incorporating spatial zoning policies and field-based biodiversity surveys. Furthermore, incorporating higher-resolution multi-source data and integrating field-based verification would improve the reliability and accuracy of the findings. These improvements would provide more comprehensive and precise scientific evidence to better support ecological protection and sustainable development in Xinjiang.

## 5. Conclusions

(1) The overall level of FVC in Xinjiang is comparatively low and exhibits a distinct north–south gradient. Vegetation cover in the north is higher than in the south, with the Tianshan mountains serving as a natural boundary. From 2000 to 2024, the annual mean FVC in Xinjiang showed fluctuations but generally increased, reaching a maximum of 0.241, a minimum of 0.215, and an average of 0.226.

(2) From 2000 to 2024, the overall vegetation cover in Xinjiang experienced steady improvement. The proportion of lowest and low vegetation cover areas decreased by 2.71%, while the proportion of high and highest vegetation cover areas increased by 2.35%. Specifically, the area of lowest vegetation cover regions declined from approximately 8.79 × 10^5^ km^2^ in 2000 to about 08.62 × 10^5^ km^2^ in 2024. In contrast, the area of highest vegetation cover regions expanded from approximately 1.60 × 10^5^ km^2^ in 2000 to about 1.89 × 10^5^ km^2^ in 2024.

(3) Between 2000 and 2024, vegetation cover in Xinjiang showed a pattern of “coexistence of improvement and degradation”. Areas with basically unchanged vegetation cover accounted for the largest proportion at 50%. Areas with significant increases accounted for 10%, and those with significant decreases accounted for 9%. Overall, despite notable achievements in ecological restoration and a generally positive trend, the risk of ecological degradation in certain localized areas persists as a challenge.

(4) The CV for vegetation cover in Xinjiang exhibited significant temporal differentiation. The proportion of areas with insignificant variation decreased from 85.46% to 69.71%, while the proportion of areas with significant fluctuations increased from 8.02% to 15.04%. These findings indicate that, over time, vegetation variability has gradually intensified, and stability has correspondingly declined.

(5) Among the 15 driving factors influencing vegetation cover in Xinjiang, the most influential natural factors include vegetation type, soil type, precipitation, and temperature, while land use type stands out as the most impactful anthropogenic factor. Of all the factors, the aspect has the least influence. The combined effect of any two factors on FVC exceeds that of individual factors, particularly the interaction between land use type and vegetation type, demonstrating significant linear enhancement. Overall, the interplay between natural and anthropogenic factors collectively shapes the spatiotemporal differentiation of vegetation cover in Xinjiang.

## Figures and Tables

**Table 1:** Source and description of driving factors.

Type	Factor	Resolution/m	Data Source
Natural	Precipitation	5000	UCSB-CHG/CHIRPS/DAILY
Natural	Temperature	1000	MOD13A2.061
Natural	Elevation	30	Copernicus DEM GLO30
Natural	Slope	30	Copernicus DEM GLO30
Natural	Aspect	30	Copernicus DEM GLO30
Natural	geomorphology	1000	Resdc
Natural	Soil type	1000	Resdc
Natural	Vegetation type	1000	Resdc
Natural	Drainage density	1000	https://cstr.cn/31253.11.sciencedb.j00001.00759 (accessed on 12 November 2024)
Natural	Distance to rivers	/	OpenStreetMap
Human	Distance to roads	/	OpenStreetMap
Human	Land use type	10	ESA/WorldCover/v100
Human	Night light	500	NPP/VIIRS
Human	Population	1000	https://landscan.ornl.gov/ (accessed on 12 November 2024)
Human	GDP	1000	Resdc

**Table 2:** The division of FVC.

Grade	FVC(%)	Landscape Performance
Highest	>70	Grassland, woodland, etc.
High	50~70	Patchy sandy land, medium- and high-yield grassland, woodland, etc.
Medium	30~50	Fixed dunes, farmland, etc.
Low	10~30	Mobile dunes, low-yield grassland, and sparse woodland, etc.
Lowest	<10	Mobile dunes, residential areas, water bodies, transportation, and construction land, etc.

**Table 3:** The division of trends by Theil–Sen and Mann–Kendall.

β	|Z|	Class
>0.0005	>2.58	Significant increase
>0.0005	<2.58	Slight increase
<−0.0005	>2.58	Significant decrease
<−0.0005	<2.58	Slight decrease
Else		Basically unchanged

**Table 4:** The division of future development trends.

Sen + M-K Result	Hurst Exponent	Future Development Trend
Increase	<0.5	Continued improvement
Increase	>0.5	Improvement to degradation
Decrease	<0.5	Continued degradation
Decrease	>0.5	Degradation to improvement
Else		Basically unchanged

**Table 5:** The division of stability by CV.

CV	Grade
0~0.15	Extremely insignificant
0.15~0.25	Insignificant
0.25~0.35	Moderate
0.35~0.5	Significant
>0.5	Extremely significant

**Table 6:** Types of interaction.

Interaction Relationship	Interaction Types
*q*(*X*_1_∩*X*_2_) < min{*q*(*X*_1_),*q*(*X*_2_)}	Nonlinear–weaken
min{*q*(*X*_1_),*q*(*X*_2_)} < *q*(*X*_1_∩*X*_2_) < max{*q*(*X*_1_),*q*(*X*_2_)}	Uni-variable–weaken
*q*(*X*_1_∩*X*_2_) > max{*q*(*X*1),*q*(*X*_2)_}	Bivariable–enhanced
*q*(*X*_1_∩*X*_2_) = *q*(*X*_1_) + *q*(*X*_2_)	Independent
*q*(*X*_1_∩*X*_2_) > *q*(*X*_1_) + *q*(*X*_2_)	Nonlinear–enhanced

**Table 7:** Changes in Xinjiang vegetation cover levels from 2000 to 2024.

FVC Grade	Area Percentage					
	2000	2006	2012	2018	2024	Change
Lowest	53.91%	52.30%	48.47%	52.15%	52.89%	−1.03%
Low	25.61%	26.61%	28.74%	25.15%	23.93%	−1.68%
Medium	6.42%	6.46%	7.15%	6.82%	6.78%	0.36%
High	4.25%	4.35%	4.69%	4.71%	4.80%	0.55%
Highest	9.80%	10.28%	10.96%	11.18%	11.60%	1.80%

**Table 8:** Similarity assessment metrics (MAE, RMSE, R², SSIM, PSNR) of FVC across validation years.

Year	MAE	RMSE	R^2^	SSIM	PSNR
2000	0.7703	0.7356	0.7820	0.4539	11.55
2006	0.9976	0.9512	0.4966	0.9835	26.23
2012	0.9976	0.9511	0.4966	0.9834	26.11
2018	0.9976	0.9512	0.4966	0.9835	26.24

**Table 9:** Changes in the stability of vegetation cover from 2000 to 2024.

Stability Grade	Area Percentage					
	2000–2006	2007–2012	2013–2018	2019–2024	Change	Mean
Extremely insignificant	62.91%	53.57%	53.56%	37.51%	−25.39%	48.67%
Insignificant	22.55%	27.52%	27.83%	32.20%	9.64%	33.50%
Moderate	6.53%	9.24%	8.48%	15.25%	8.72%	8.73%
Significant	3.37%	4.69%	4.81%	7.72%	4.35%	4.51%
Extremely significant	4.65%	4.98%	5.31%	7.32%	2.68%	4.59%

**Table 10:** CV of FVC from 2000 to 2024 based on the five ecological sub-regions in Xinjiang.

Zone	Extremely Insignificant	Insignificant	Moderate	Significant	Extremely Significant
I	55.54%	33.39%	6.52%	2.16%	2.39%
II	48.04%	33.17%	10.24%	4.79%	3.77%
III	52.00%	31.56%	7.73%	3.19%	5.53%
IV	24.24%	41.17%	15.60%	10.17%	8.82%
V	21.54%	44.94%	14.35%	6.40%	12.77%

**Table 11:** Top three interactive factor combinations with land use type across ecological sub-regions in Xinjiang. (*X* represents the factor interacting with land use type, and *q*-max denotes the explanatory power after interaction).

	*x*_1_	*q*-max_1_	*x*_2_	*q*-max_2_	*x*_3_	*q*-max_3_
I	Temperature	0.731	Vegetation	0.724	Soil Type	0.688
II	Population	0.785	Vegetation	0.777	Temperature	0.770
III	Vegetation	0.710	Soil Type	0.689	Temperature	0.664
IV	Population	0.788	Vegetation	0.787	Temperature	0.783
V	Elevation	0.570	Population	0.560	GDP	0.549

## Data Availability

The FVC products and code utilized in this study are publicly accessible at https://github.com/2861707601/FVC_XinJiang/tree/main (accessed on 12 November 2024).
